# A process-oriented measure of habit strength for moderate-to-vigorous physical activity

**DOI:** 10.1080/21642850.2014.896743

**Published:** 2014-03-31

**Authors:** J. Robert Grove, Irja Zillich, Nikola Medic

**Affiliations:** ^a^ School of Sport Science, Exercise, and Health (M408), University of Western Australia, 35 Stirling Highway, Crawley, WA6009, Australia; ^b^ Department of Psychology, University of Mainz, Binger Strasse 14-16, 55122Mainz, Germany; ^c^ School of Exercise and Health Sciences, Edith Cowan University, 270 Joondalup Drive, Joondalup, WA6027, Australia

**Keywords:** health behaviour, exercise, physical activity, habits, routines

## Abstract

*Purpose*: Habitual action is an important aspect of health behaviour, but the relevance of various habit strength indicators continues to be debated. This study focused specifically on moderate-to-vigorous physical activity (MVPA) and evaluated the construct validity of a framework emphasizing patterned action, stimulus-response bonding, automaticity, and negative consequences for nonperformance as indicators of habit strength for this form of exercise. *Methods*: Upper-level undergraduates (*N* = 124) provided demographic information and responded to questionnaire items assessing historical MVPA involvement, current MVPA involvement, and the four proposed habit strength dimensions. Factor analyses were used to examine the latent structure of the habit strength indicators, and the model's construct validity was evaluated via an examination of relationships with repetition history and current behaviour. *Results*: At a measurement level, findings indicated that the proposed four-component model possessed psychometric integrity as a coherent set of factors. Criterion-related validity was also demonstrated via significant changes in three of the four factors as a function of past involvement in MVPA and significant correlations with the frequency, duration, and intensity of current MVPA. *Conclusions*: These findings support the construct validity of this exercise habit strength model and suggest that it could provide a template for future research on how MVPA habits are developed and maintained.

Habit development is important for obtaining maximum benefits from any health-related behaviour. It is well known that the typical pattern of change for such behaviours consists of a progression from occasional, irregular performance to more frequent and regular involvement, and that repetition over an extended period of time is needed to improve the chances of maintaining the new regime (Prochaska & Velicer, 1997; Prochaska et al., 1994). In part, this is because the initiation of new behaviours requires deliberation and conscious effort, but, after the behaviour has been repeated many times, it requires less conscious effort. Therefore, it can be performed in a semi-automatic or habitual fashion and becomes more resistant to change (Aarts & Dijksterhuis, 2000; Ajzen & Fishbein, 2000; Bargh & Chartrand, 1999).

Given the central role that habits play in the maintenance of health behaviour, it is important to have a thorough understanding of how habits can be assessed, both in a general sense and in a behaviour-specific sense. Indeed, the identification and measurement of processes that support habit development is a crucial aspect of designing and implementing effective interventions to improve health status (Velicer, Rossi, Prochaska, & Diclemente, 1996). Unfortunately, this has proven to be a difficult undertaking for a variety of reasons (Ajzen, 2002). Although there is general consensus that cue-based automaticity is a central feature of health-related habits (Gardner, Abraham, Lally, & de Bruijn, 2012a; Orbell & Verplanken, 2010), the relevance of other components continues to be debated at both a theoretical and measurement level (Gardner, Abraham, Lally, & de Bruijn, 2012b; Sniehotta & Presseau, 2012).

## The structure of exercise habits

A conceptual framework outlined by Grove, Jackson, Longbottom, and Medic (2013) may serve to eliminate some of this confusion with respect to one particular health-related behaviour, namely, moderate-to-vigorous physical activity (MVPA). This framework suggests that habit strength for physical activity is defined (and therefore can be assessed) by the strength of four specific psycho-behavioural processes: stimulus-response (SR) bonding, automaticity, patterning of action, and negative consequences from nonperformance. Considerable research evidence supports each of these processes as a viable indicator of habit strength. For example, the strengthening of *SR bonds* via association is a core component of learning and a key determinant of how often a behaviour is repeated (Hull, 1952). When these bonds become sufficiently strong, the behaviour in question can be directly triggered by environmental cues that are associated with the activity (Bargh & Chartrand, 1999). In other words, a key feature of habitual responses is that they are set in motion when one encounters people, places, or other activities that have consistently been associated with that behaviour in the past (Wood & Neal, 2007).

*Automaticity* is also widely acknowledged as a core element of habitual behaviour (Aarts & Dijksterhuis, 2000; Bargh & Chartrand, 1999; Gardner et al., 2012a; Orbell & Verplanken, 2010). With increasing amounts of repetition, behaviours become more well learned; they make fewer immediate demands on cognitive capacities; and they can be executed at lower levels of conscious awareness (Ajzen, 2002; Ajzen & Fishbein, 2000; Wood, Quinn, & Kashy, 2002). This shift towards automated execution occurs quite naturally as a function of repetition, and it has been demonstrated for a variety of complex behaviours, including physical activity involvement (Aarts, Paulussen, & Schaalma, 1997; Bargh & Chartrand, 1999; Lally, van Jaarsveld, Potts, & Wardle, 2010; Verplanken & Melkevik, 2008).

*Response patterning* as a function of repetition has considerable support in both the motor learning literature and the social psychology literature. More specifically, repeated practice is associated with reduced variability for discrete movements (Giuffrida, Shea, & Fairbrother, 2002; Lay, Sparrow, Hughes, & O'Dwyer, 2002), and the motor programmes governing complex movements also become increasingly invariant with more and more repetition (Schmidt, 2003; Shea & Wulf, 2005). These findings are consistent with the social psychological view that repetition of macro-level behavioural repertoires leads to procedural encoding, heuristic processing, and scripting which, in turn, results in an integrated, patterned, and predictable action sequence that is resistant to change (Anderson, 1982; Chen & Chaiken, 1999; Ewart, 1991).

Similarly, there is strong evidence that *negative psychological consequences* are experienced if consistently repeated and well-ingrained behaviours are not (or cannot be) performed. Indeed, one function of behavioural routines is to regulate emotions, and disruption of these routines has been shown to produce substantial increases in stress levels (DeCaro & Worthman, 2011; Lawson, Waller, & Lockwood, 2007; Luo & Cooper, 1990). Importantly, restriction of physical activity among regular exercisers produces a number of well-documented psychological distress symptoms that include guilt, irritability, anxiety, depression, and feelings of loss (Berlin, Kop, & Deuster, 2006; Mondin et al., 1996; Poole, Hamer, Wawrzyniak, & Steptoe, 2011; Weinstein, Deuster, & Kop, 2007).

## The current study

On the basis of this evidence, and using MVPA as a frame of reference, the purposes of this study were to: (a) generate coherent, multiple-item measures of the four processes proposed by Grove et al. (2013) as indicators of habit strength for physical activity (i.e. strength of SR bonds, automaticity, patterning of action, and negative consequences for nonperformance) and (b) evaluate the criterion-related validity of these measures by assessing the extent to which they change as a function of MVPA history and the extent to which they correlate with current MVPA involvement.

## Method

### Participants and general procedure

Following institutional ethics approval, upper-level undergraduates at a mid-sized university were invited to participate in “a study on exercise attitudes and behaviour”. A total of 124 students agreed to participate and signed an informed consent form before completing a four-part questionnaire. The sample consisted of 25 males and 99 females, with an average age of 21.9 years (SD = 4.8 years).

### Questionnaire measures

The first section of the questionnaire requested standard *demographic information* (age, gender, etc.), and the second section asked respondents to classify themselves into one of five categories based on their *history of MVPA involvement* during the past six months (Cardinal, 1995). These categories corresponded to the precontemplation, contemplation, preparation, action, and maintenance stages of change (Prochaska & DiClemente, 1984). The general frame of reference for stage-of-change classification was

During the past six months, how frequently were you involved in physical activities that made your heart beat faster than normal, or that made you hot and sweaty, or that made you huff and puff? Examples include brisk walking, jogging, cycling, swimming, and vigorous sports. (cf. Godin & Shephard, 1985)

Specific response options were as follows: “I haven't done that type of activity, and I don't plan to start in the next month” (precontemplation); “I haven't done that type of activity, but I am thinking about starting sometime soon” (contemplation); “I have occasionally done that type of activity, but not on a regular basis” (preparation); “I have been doing that type of activity on a regular basis, but I started less than six months ago” (action); and “I have been doing that type of activity on a regular basis for six months or more” (maintenance).

The third section of the questionnaire assessed the frequency, duration, and intensity of *current involvement in MVPA*. The frame of reference for these responses was the same as that in the second section of the questionnaire (i.e. “physical activities that make your heart beat faster than normal, or that make you hot and sweaty, or that make you huff and puff”). Frequency was assessed by asking “On average, how many days per week do you currently engage in this type of physical activity?”, with responses made on an eight-point scale ranging from never (0) to every day (7). Responses to the duration question (“On average, how long does each physical activity session last?”) were recorded in minutes, and they were subsequently combined with the frequency information to determine minutes per week of physical activity. Responses to the intensity question (“On average, what is the intensity of these physical activity sessions?”) reflected continuous points on the Borg Rating of Perceived Exertion (RPE) scale (Borg, 1982) and ranged from “very, very light” (7) to “very, very hard” (19).

The fourth section of the questionnaire included 27 items designed to assess the four processes identified by Grove et al. (2013) as *indicators of habit strength for physical activity* (i.e. automaticity, SR bonds, patterning of action, and negative consequences for nonperformance). Some of these items were adapted from existing measures of action−awareness merging, commitment to physical activity, and exercise dependence (Corbin, Nielsen, Borsdorf, & Laurie, 1987; Grove & Lewis, 1996; Jackson, 1995; Ogden, Veale, & Summers, 1997; Verplanken & Orbell, 2003), while others were written specifically for this study. Examples included “I exercise without having to think about it” (automaticity), “Certain surroundings just make me want to exercise” (SR bonds), “Most of my exercise sessions follow the same pattern” (patterning of action), and “If I don't exercise I feel restless” (negative consequences). Participants were again instructed to use “physical activities that make your heart beat faster than normal, or that make you hot and sweaty, or that make you huff and puff” as a frame of reference for their responses, which were made on a six-point bipolar scale anchored by “not true for me” (1) and “very true for me” (6).

### Data analyses

Data analysis consisted of: (a) an exploratory factor analysis (EFA) to identify the latent factor structure of the habit process items; (b) calculation of descriptive statistics, with a specific focus on the Borg RPE scores to confirm that responses reflected MVPA; (c) an assessment of construct validity for the habit strength indicators based on changes as a function of repetition history (i.e. stage-of-change); and (d) additional construct validity analyses based on relationships between the habit strength indicators and current involvement in MVPA.

## Results

### Habit strength indicators

Examination of descriptive statistics for the 27 habit process items indicated no distributional concerns for any of the items (all skewness values less than |1.04| and all kurtosis values less than |1.40|). The items also demonstrated satisfactory interitem dependence (*χ* ^2^ = 993.01, *p* < 0.001) and an acceptable Kaiser-Meyer-Olkin (KMO) sampling adequacy statistic (KMO = 0.77), confirming suitability of the interitem correlation matrix for factor analysis (Dziuban & Shirkey, 1974).

The latent factor structure of the item pool was therefore examined using an iterative, EFA approach (Gerbing & Hamilton, 1996). More specifically, we conducted principal-axis factor analysis with direct oblimin transformations (*δ* = −1) using Thurstone's simple structure criteria, factor interpretability, and factor definition as the criteria for item retention (Russell, 2002). Following joint consideration of the Kaiser–Guttman (eigenvalues >1) and scree plot stopping rules (Cattell, 1978), a four-factor solution was determined viable and pursued. These analyses resulted in the retention of 17 items that accounted for 63.7% of the overall variance. Examination of the transformed pattern matrix (shown in Table 1) indicated adequate simple structure (i.e. all loadings >|0.45| on one factor and <|0.25| on other factors), and inspection of subscale content revealed conceptual clarity with respect to the assessment of negative consequences for nonperformance, patterning of action, strength of SR bonds, and automaticity. Cronbach's alphas (also shown in Table 1) indicated good internal consistency for all four factors, with values ranging from 0.77 to 0.85.

**Table 1.** Latent factor structure for the MVPA habit strength indicators.

	NEG	PAT	SRB	AUT
*Negative consequences if not done (NEG)*				
(h1) If I don't exercise I feel restless	.**87**	.18	−.00	−.01
(h2) If I don't exercise I feel tense	.**70**	−.03	−.16	.01
(h3) If I don't exercise I feel tired	.**62**	−.18	.02	.03
(h4) If I don't exercise I feel irritable	.**61**	.16	−.06	.06
*Patterned action (PAT)*				
(h5) I exercise for the same amount of time in each session	.01	.**79**	−.06	−.13
(h6) I tend to do the same activities or exercises in each session	.02	.**76**	−.08	−.17
(h7) Most of my exercise sessions follow the same pattern	−.06	.**71**	−.15	−.03
(h8) I exercise on the same days each week	.06	.**52**	.09	.24
(h9) I exercise at the same location each week	.03	.**46**	.05	.18
*Strong SR bonds (SRB)*				
(h10) Seeing other people exercise motivates me to be more active	−.03	.08	**−**.**72**	.10
(h11) When I see someone else exercising, I feel like exercising	−.05	.05	**−**.**61**	.09
(h12) Some situations give me a desire to exercise	.04	−.05	**−**.**82**	−.02
(h13) Certain surroundings just make me want to exercise	.18	−.02	**−**.**79**	−.06
*Automaticity (AUT)*				
(h14) I exercise without having to think about it	.08	.18	.02	.**73**
(h15) I exercise spontaneously and automatically	.04	.10	−.18	.**65**
(h16) I attend exercise sessions without conscious thought	.21	.08	−.04	.**51**
(h17) I exercise without conscious reminders to do so	−.01	−.14	−.01	.**48**
Eigenvalue	5.45	2.38	1.91	1.09
Percent variance	32.06	13.99	11.21	6.44
Cronbach's alpha	.84	.78	.85	.77

### Current involvement in MVPA

Descriptive statistics for the habit strength indicators and the self-reported frequency, intensity, and duration of current physical activity are given in the first two columns of Table 2. Importantly, the mean intensity rating for the activities undertaken by the participants was 13.39 on the Borg scale, which confirms that their responses were indeed reflective of MVPA involvement according to RPE criteria outlined by Norton, Norton, and Sadgrove (2010). Examination of the other descriptive information indicated that, overall, the participants were living a moderately active lifestyle, with approximately 3 × 45 minutes of MVPA per week.

**Table 2.** Descriptive statistics and correlations of the habit strength indicators with MVPA measures.

Measure	Mean	SD	NEG	PAT	SRB	AUT	TOT
Negative consequences (NEG)	3.58	1.16					
Patterned action (PAT)	3.92	1.04	.26				
SR bonds (SRB)	4.45	0.98	.56	.35			
Automaticity (AUT)	2.66	0.96	.53	.33	.33		
Habit strength total (TOT)	14.59	2.98	.81	.65	.76	.73	
Activity intensity (Borg RPE scale)	13.38	1.80	.26**	.15	.12	.09	.24*
Activity frequency (days/week)	2.99	1.43	.23*	.12	.24*	.40***	.36***
Activity duration (minutes/session)	46.93	20.37	.15	.22*	.07	.37***	.29***
Weekly minutes (sessions×minutes/session)	140.32	109.63	.26**	.20*	.20*	.50***	.42***

*p < 0.01.

**p < 0.01.

***p < 0.001.

### Construct validity evidence

**Figure 1.**
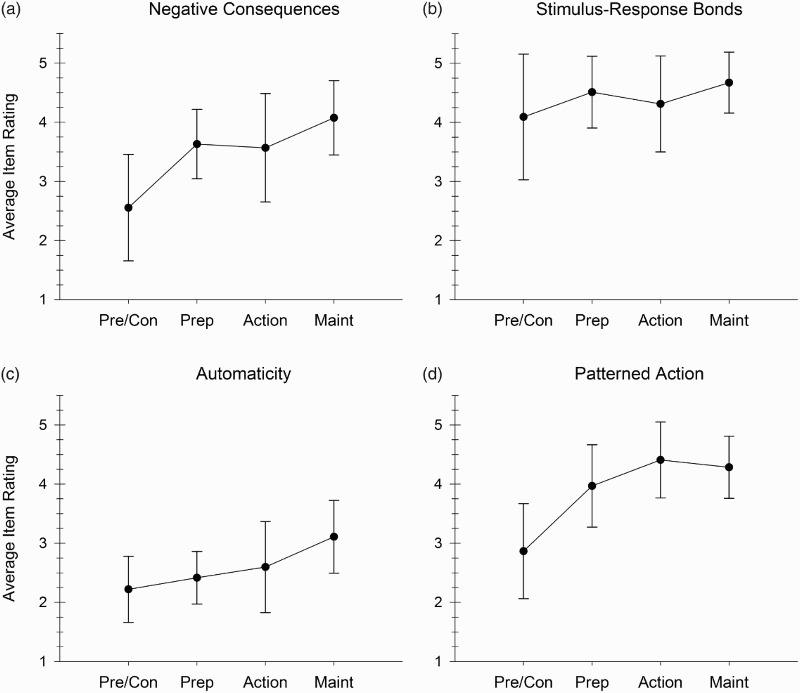
Adjusted means for the habit strength indicators in relation to stage-of-change for MVPA (estimated marginal mean ±95% confidence interval; covariate = current frequency of exercise).

Two sets of analyses addressed the construct validity of the MVPA habit strength indicators given in Tables 1 and 2. First, multivariate analysis of covariance (MANCOVA) procedures were used to determine whether scores on these indicators differed as a function of the individual's repetition history after controlling for frequency of current exercise. If these processes are valid indicators of habit strength for MVPA, then scores for individuals with a history of regular MVPA involvement (i.e. those in the later stages of change) should be substantially higher than those with minimal or sporadic MVPA involvement (i.e. those in the earlier stages of change). Overall, findings from the analysis suggested that this was indeed the case. More specifically, a MANCOVA with current exercise frequency employed as the covariate revealed a significant multivariate effect for stage-of-change, Wilks' lambda = 0.648, *F* (12, 296) = 4.41, *p* < 0.001, with subsequent examination of univariate results confirming significant stage-related differences on three of the four subscales [*F* (3, 115) = 7.94, *p* < 0.001 for patterning of action; *F* (3, 115) = 5.94, *p* = 0.001 for negative consequences; and *F* (3, 115) = 4.36, *p* = 0.006 for automaticity]. The nature of these effects can be seen in Figure 1, which reveals a general increase in the strength of these three processes across the stage-of-change continuum. *Post hoc* comparisons using *Bonferroni* corrections indicated that participants in the preparation, action, and maintenance groups had significantly higher scores than those in the precontemplation/contemplation group on patterned action and negative consequences (*p* < 0.05). For automaticity, the precontemplation/contemplation, preparation, and action groups were not significantly different, but the maintenance group exhibited significantly higher scores than either the precontemplation/contemplation group or the preparation group (*p* < 0.05).

The second set of construct validity analyses was undertaken using Pearson correlations to investigate relationships between self-reported frequency, duration, and intensity of current weekly MVPA and scores on the four habit strength indicators (as well as a total habit strength score created by summing the individual subscale scores). Positive correlations with these self-report measures would be expected if the individual habit process measures and the composite score are indicative of habit strength for MVPA. Once again, results suggested that this was the case, and the findings are summarized in the bottom portion of Table 2. Inspection of these values indicates that automaticity, patterned action, SR bonds, negative consequences for nonperformance, and the total habit strength score were all positively related to current exercise frequency as well as the average duration and intensity of the current exercise sessions. In addition, when the frequency and duration data were combined to produce a minutes-per-week (“weekly minutes”) measure, that index was positively and significantly correlated with all four subscales and the total habit strength score.

## Discussion

Habits exert a direct effect on health behaviour, and the magnitude of this effect is comparable to that of cognitive, motivational, and affective factors (de Bruijn, Kremers, Singh, van den Putte, & van Mechelen, 2009; Chatzisarantis & Hagger, 2007; Gardner, de Bruijn, & Lally, 2011; Rhodes, de Bruijn, & Matheson, 2010). The identification and measurement of processes that support habit development is therefore a crucial aspect of designing and implementing effective interventions to improve health status (Velicer et al., 1996). In this study, we focused specifically on MVPA and evaluated the construct validity of a four-component framework that emphasizes patterning of action, strength of SR bonds, automaticity, and negative consequences for nonperformance as indicators of habit strength for this form of exercise. A detailed understanding of the processes surrounding habitual MVPA is particularly important because of the central role this behaviour plays in physical and mental health across the lifespan (Haskell et al., 2007; Pavey, Peeters, Bauman, & Brown, 2013).

Findings indicated that the elements of this process-oriented model: (a) can be assessed economically via coherent clusters of questionnaire items (Table 1); (b) have psychometric integrity as an inter-related yet non-redundant set of factors (Table 2, top panel); (c) display a general tendency to increase in strength as a consequence of one's past history of involvement in MVPA (Figure 1); and (c) correlate positively with the frequency, duration, and intensity of current MVPA (Table 2, bottom panel). Together, these findings support the construct validity of this exercise habit strength model and suggest that it could provide a template for future research on how exercise habits are developed and maintained.

For example, it is logical to assume that imposing a structure or pattern on initial MVPA involvements will stabilize the cues associated with those behaviours and, over time, strengthen the associated SR bonds. The strengthening of SR bonds may, in turn, lead to increased automaticity for MVPA. Once automaticity has been established, nonperformance of the MVPA behaviours may generate negative consequences, with the strength of those negative consequences perhaps influenced by the degree to which automaticity has been developed. Such a proposed sequence is, of course, speculative at this point and would require the use of longitudinal research designs to be adequately evaluated. However, it is entirely consistent with behavioural approaches that emphasize alterations in how activities are undertaken as an initial step in behaviour change (Foster, Makris, & Bailer, 2005; Martin & Dubbert, 1982; Wing, 2002). It is also consistent with theoretical and empirical work on the associations between cueing, automaticity, and negative affect in connection with various health behaviours (Hashim, Jawis, Wahat, & Grove, 2014; Orbell & Verplanken, 2010; Tappe & Glanz, 2013; Wood & Neal, 2007). As such, it is certainly worthy of investigation in future studies.

At a more practical level, it is noteworthy that all four of the habit strength indicators given in Table 1 and Figure 1 can be identified within interventions known to be effective in increasing the frequency of exercise behaviour. For example, a “same activity, same time, same place” (i.e. behavioural patterning) strategy has been shown to have beneficial effects on exercise frequency and exercise adherence (Dubbert, Rappaport, & Martin, 1987). Similarly, the strengthening of activity-specific SR bonds via point-of-decision prompts and other stimulus control procedures also has a positive influence on exercise activity (Kahn et al., 2002; Marcus, Rossi, Selby, Niaura, & Abrams, 1992). Active transport strategies encourage automaticity of energy expenditure by deliberately linking exercise with frequently undertaken and therefore highly automated daily activities (de Bruijn et al., 2009; Gordon-Larsen, Nelson, & Beam, 2005; Grow et al., 2008; Morency & Demers, 2010). Negative consequences for nonperformance are an element of self-reevaluation and environmental reevaluation, two important processes of change within the transtheoretical model (Grove et al., 2013; Marcus et al., 1992). They are also the defining feature of interventions that emphasize anticipated regret, and these interventions have been shown to positively influence a wide range of health behaviours including exercise (Abraham & Sheeran, 2003; Conner & Abraham, 2001; Sandberg & Conner, 2008). Collectively, these links to existing intervention practices support the ecological validity of the model presented here, and they also suggest that it might help to satisfy a need for the identification of specific behaviour change strategies that will facilitate the development of positive health habits (Gardner et al., 2012b).

## Perspectives, limitations, and future directions

The MVPA habit model addressed in this study includes elements that could be viewed as developmental antecedents (i.e. patterning of action), core processes (strong SR bonds and automaticity), and maintenance factors (i.e. negative consequences). Other researchers have addressed similar elements in prior work on health-habits (Orbell & Verplanken, 2010; Tappe & Glanz, 2013; Wood & Neal, 2007), and some have argued that particular elements should be given more consideration than others (e.g. automaticity; Gardner et al., 2012a; Sniehotta & Presseau, 2012). With respect to MVPA, our findings suggest that the broader perspective has merit and may provide guidance on how habits connected to this important form of exercise can be assessed, developed, and maintained. At the same time, it must be acknowledged that the evidence presented here was obtained exclusively from self-reports. As such, there is a need for corroboration of these findings in studies that employ implicit assessments of MVPA habit strength and/or objective measures of MVPA behaviour. It must also be acknowledged that the frame of reference for our research was specific to MVPA, and different processes might be associated with the habitual undertaking of other types of exercise (e.g. regular walking). We therefore encourage our colleagues to conduct further examinations of this framework in connection with various forms of exercise. We also encourage them to explore the potential relevance of the framework to health behaviours other than exercise where habit strength might be reflected by processes similar to those examined here.
